# Differential responses of post-exercise recovery leg blood flow and oxygen uptake kinetics in HFPEF versus HFREF

**DOI:** 10.1186/1532-429X-18-S1-O9

**Published:** 2016-01-27

**Authors:** Richard B Thompson, Joseph J Pagano, Ian Paterson, Jason Dyck, Dalane Kitzman, Mark Haykowsky

**Affiliations:** 1 grid.17089.37Biomedical Engineering, University of Alberta, Edmonton, AB Canada; 2 grid.267315.40000000121819515College of Nursing and Health Innovation, University of Texas at Arlington, Arlington, TX USA; 3 grid.17089.37Medicine, University of Alberta, Edmonton, AB Canada; 4 grid.17089.37Pediatrics, University of Alberta, Edmonton, AB Canada; 5 grid.241167.70000000121853318Cardiology and Geriatrics, Wake Forest University, Wake Forest, NC USA

## Background

Delayed oxygen uptake (VO_2_) kinetics during recovery from a bout of endurance exercise have been shown to be an important prognostic marker of all-cause mortality in chronic heart failure (HF), where skeletal muscle is the predominant O_2_ consumer. Few studies have examined skeletal muscle O_2_ delivery/utilization, and no previous study has evaluated the differences between HF patients with reduced LVEF (HFREF) versus those with preserved LVEF (HFPEF). We used novel MRI-based techniques to non-invasively measure quadriceps (leg) blood flow, O_2_ extraction and VO_2_ recovery kinetics in clinically stable patients diagnosed with HFREF or HFPEF.

## Methods

**Figure 1:**
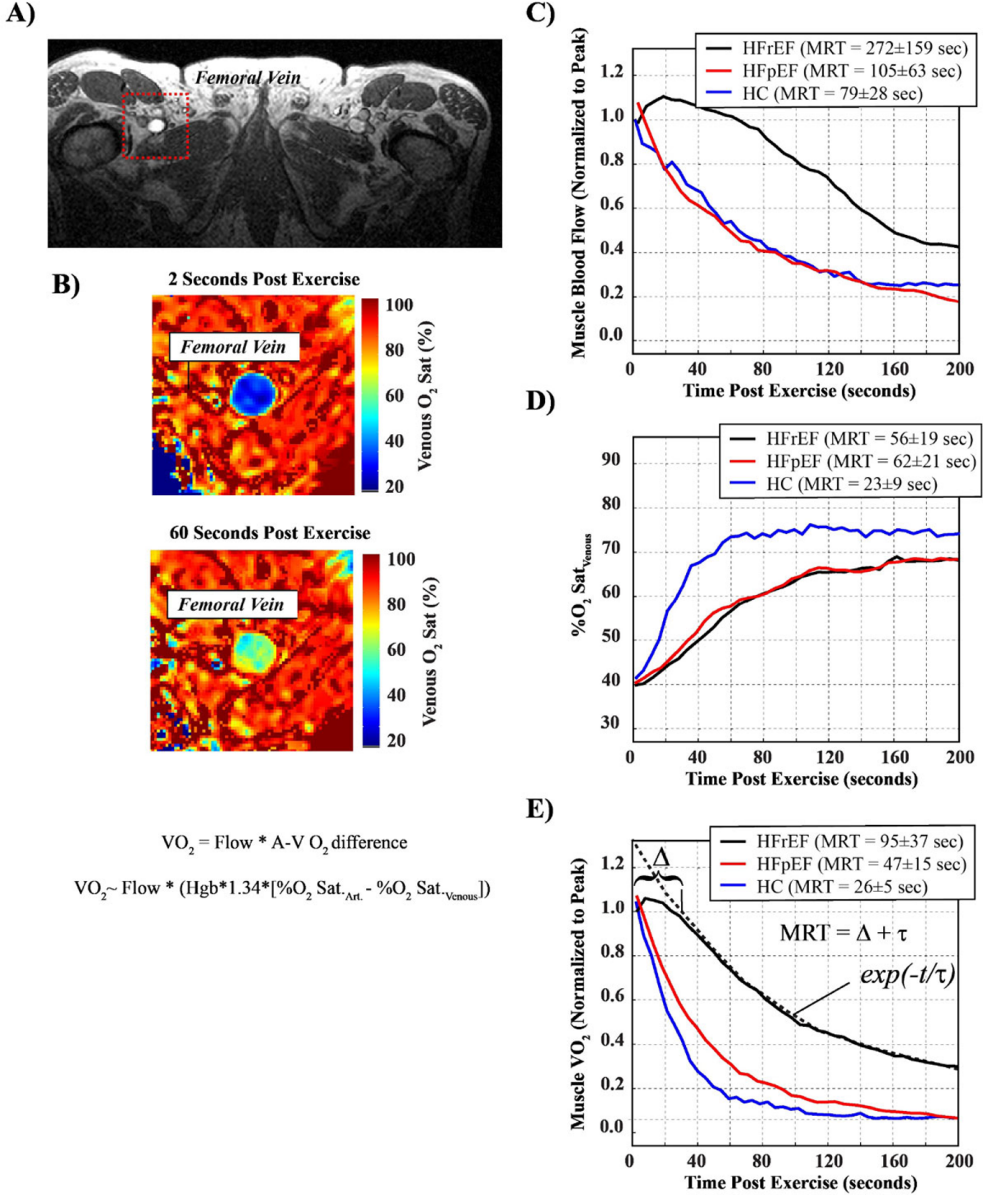
**A)** Anatomic image from a patient showing femoral vein location used for evaluation of flow and venous O2 saturation. **B)** O2 saturation images from a patient at two time points (2 sec. and 60 sec.) following exercise, and the Fick equation for calculation of VO2. **C)** to **E)** show the average recovery curves for flow, venous O2 saturation and calculated leg VO2, for HFPEF (black), HFPEF (red) and healthy controls (HC, blue). MRT = mean response time, which is the sum of the delay term (Δ), to the onset of exponential recovery, and time constant of the best-fit mono-exponential decay function (t), as shown in **E)**. HFPEF = heart failure with preserved ejection fraction, HFREF = heart failure with reduced ejection fraction, Hgb = hemoglobin concentration.

Leg flow and venous O_2_ saturation (%SaO_2_) were measured in the femoral vein post-exercise (knee-extension) using MRI (Fig. 1A, B) as previously described (Magn Reson Med. 2014 Dec 22. doi: 10.1002/mrm.25564). These values in conjunction with arterial oxygen saturation (%SaO_2_, pulse oximeter), hemoglobin (Hgb) and hematocrit (from blood sampled prior to exercise) are used to calculate leg VO_2_, from the Fick equation (Fig. 1B). All subjects performed 4 min. of single-leg knee-extension exercise at 85% of their pre-determined peak power output. Leg blood flow, oxygen extraction and VO_2_ were measured continuously during recovery for 3 minutes, starting within 1 second of exercise cessation. Recovery kinetics were quantified as the mean response time (MRT - defined in Fig. 1E, lower right panel) for all parameters, with comparison to healthy younger male controls (HC) from a previous study using the same methodology.

## Results

HFPEF (n = 5, LVEF = 36 ± 11%, 69 ± 9 yrs) and HFPEF (n = 5, LVEF = 57 ± 6%, 67 ± 11 yrs) patients were recruited from the Alberta HEART study. Quadriceps muscle mass, peak leg flow, A-VO_2_ difference and VO_2_ were not significantly different between HFPEF and HFREF (p > 0.05 for all). However, HFREF patients had severe impairment of VO_2_ recovery kinetics (increased MRT)_,_ while HFPEF had a moderate impairment, as compared to HC (p < 0.05 for all comparison, Fig. 1E, bottom right). This is understood by considering the underlying flow and oxygen extraction kinetics. From Fig. 1D) both HF groups showed similarly impaired A-VO_2_ recovery kinetics compared to controls (p < 0.05), however, the HFREF group had marked impairment in leg blood flow recovery dynamics, compared to both HFPEF and control groups (p < 0.05 for both comparisons, Fig. 1C). Thus, it is the impaired recovery of flow in HFREF group which distinguishes the HFREF and HFPEF groups.

## Conclusions

Whole body VO_2_ recovery kinetics are related to the degree of functional impairment and are strongly predictive of mortality. We show for the first time that muscle-specific VO_2_ recovery kinetics are significantly more delayed in HFREF compared to HFPEF (reflecting a larger oxygen debt for a similar amount of work). These findings suggest distinct mechanisms may underlie the reduced exercise capacity in HFREF vs HFPEF, with potentially distinct diagnostic metrics and therapeutic approaches.

